# Terpenoids from *Abies holophylla* Attenuate LPS-Induced Neuroinflammation in Microglial Cells by Suppressing the JNK-Related Signaling Pathway

**DOI:** 10.3390/ijms22020965

**Published:** 2021-01-19

**Authors:** Lalita Subedi, Silvia Yumnam

**Affiliations:** College of Pharmacy, Gachon University, #191, Hambakmoero, Yeonsu-gu, Incheon 21936, Korea; subedilali@gmail.com

**Keywords:** *Abies holophylla*, terpenoids, neuroinflammation, microglia, LPS, JNK, TNF-α, interleukins

## Abstract

We have previously reported that phytochemicals from *Abies holophylla* exhibit anti-inflammatory and neuroprotective effects by decreasing nitrite production and increasing nerve growth factor production. However, the exact mechanism underscoring these effects has not been revealed. In the present study, we aimed to explore the underlying anti-inflammatory mechanisms of *A. holophylla* and its phytochemicals. We studied various solvent fractions of *A. holophylla* and found the chloroform and hexane sub-fractions showed the most significant anti-neuroinflammatory effects in lipopolysaccharide (LPS)-activated murine microglia. Concomitantly, the terpenoids isolated from chloroform and hexane fractions showed similar anti-neuroinflammatory effects with significant inhibition of NO and reactive oxygen species production, and decreased protein expression of inducible nitric oxide synthase (iNOS) and cyclooxygenase. Interestingly, these terpenoids inhibited the phosphorylation of c-Jun *N*-terminal kinase (JNK), which further inhibited the production of pro-inflammatory mediators, including prostaglandin E2, tumor necrosis factor, and interleukins (IL-6 and IL-1β), with a potency greater than that of the well-known iNOS inhibitor NG-mono-methyl-L-arginine (L-NMMA). These results suggest that the chloroform- and hexane-soluble fraction mediated the mitogen-activated protein kinase (MAPK) inhibition, in particular the JNK pathway, thereby lowering the inflammatory cascades in LPS-activated murine microglia. Thus, our study suggests that the chloroform and hexane fractions of *A. holophylla* and their terpenoids may be potential drug candidates for drug discovery against LPS-induced neuroinflammation and neuroinflammatory-related neurodegeneration.

## 1. Introduction

Neuroinflammation is the chronic inflammation of the brain tissues, as the name suggests. It is associated with various neurodegenerative disorders, including Parkinson’s disease (PS) and Alzheimer’s disease (AD) [1,2,3]. Microglia, the innate immune cells of the brain, play a major role in host defense and tissue repair [4]. Although normal activation of glial cells, such as microglia and astrocytes, is responsible for maintaining normal homeostasis, excessive activation of microglia releases various pro-inflammatory mediators (nitric oxide (NO), prostaglandin E2 (PGE2)) and cytokines (interleukins (ILs) and tumor necrosis factor alpha (TNF-α)) that results in secondary damage causing neuroinflammation [4,5,6]. Moreover, chronic activation of microglia results in destabilization of the central nervous system (CNS) and neuronal degradation. Transcriptional activity of nuclear factor-kappa B (NF-κB) regulates the release of these pro-inflammatory mediators. NF-kB-mediated transcription is regulated by mitogen-activated protein kinase (MAPK) signaling. MAPKs include subgroups p38, c-Jun *N*-terminal kinase (JNK), and p42/44 extracellular signal-regulated kinase (ERK). Additionally, MAPKs are involved in the pathogenesis of inflammation of several organs, including that of the brain tissues. Activation of all or even one of the MAPKs is sufficient to induce nuclear translocation of transcription factors, leading to increased transcription of the inflammatory mediators. Furthermore, several stress types are involved in the induction of neuroinflammation. Protein kinases, particularly stress-activated protein kinase/c-Jun *N*-terminal kinase (SAPK/JNK), are activated by this stress signaling. For this reason, SAPK/JNK has garnered much attention as a key target for managing inflammatory conditions, wherein the downstream inflammatory cascades mediated by JNK signaling can be downregulated/lowered. Compounds that inhibit JNK activation reportedly exhibit beneficial effects against inflammatory bowel disease, neuroinflammation, and neurodegenerative diseases. Thus, identification of novel bioactive compounds that can modulate the release of pro-inflammatory mediators and cytokines is important in treating neuroinflammation and neurodegenerative disorders.

*Abies holophylla*, commonly known as Manchurian fir or needle fir, is widely distributed in East Asia. *Abies* species are rich sources of many bioactive secondary metabolites, such as terpenoids, lignans, flavonoids, phenols, and steroids. These compounds reportedly possess anti-inflammatory, antifungal, and antibacterial properties [7,8]. In folk medicine, *Abies* species are mainly used in the treatment of stomachache, indigestion, and vascular and pulmonary diseases [9,10]. Although we demonstrated the anti-inflammatory and neuroprotective properties of diterpenes isolated from *A. holophylla* in our previous study [11], the mechanism underlying its anti-inflammatory effect has not yet been studied.

As part of our ongoing efforts to discover bioactive secondary metabolites from *A. holophylla*, we previously conducted a bioactivity-guided isolation of the plant’s extracts and studied the anti-neuroinflammatory component present in it. In the present study, we found that chloroform- and hexane-soluble fractions from *A. holophylla* inhibited NO production in lipopolysaccharide (LPS)-activated BV2 murine microglial cells. On the basis of the bioactivity-guided isolation, sub-fractions of the chloroform- and hexane-soluble fractions were further identified and studied for their anti-inflammatory properties. The purpose of this study was to clarify the active component from *A. holophylla* subfraction that exhibits the anti-neuroinflammatory effect and its underlying mechanism, using an in vitro approach.

## 2. Results

*A. holophylla* subfractions inhibit production of NO and reactive oxygen species (ROS) and expression of inducible nitric oxide synthase (iNOS) and cyclooxygenase (COX-2) in LPS-activated microglia.

**Figure 1:**
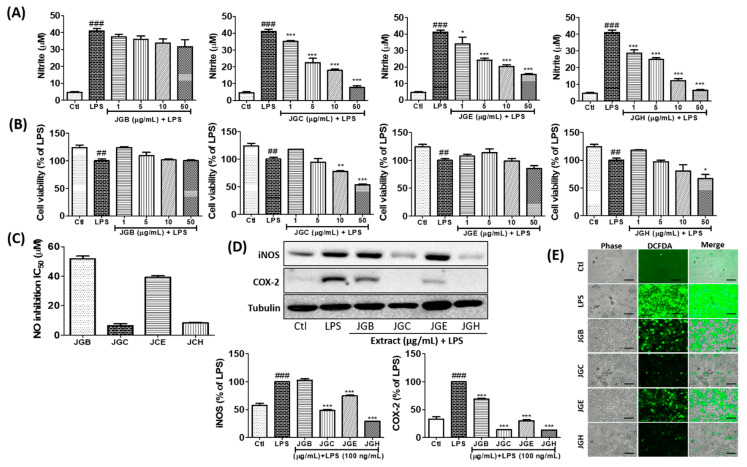
*Abies holophylla* subfractions inhibited production of inflammatory mediators and expression of inflammatory proteins. BV2 cells were pre-treated with butanol (JGB), chloroform (JGC), ethyl acetate (JGE), and hexane (JGH) fractions of *A. holophylla* for 30 min before lipopolysaccharide (LPS) (100 ng/mL) activation and further incubated for 24 h. NO was measured in conditioned medium with Griess reagent and cell viability was observed using the 3-(4,5-dimethylthiazol-2-yl)-2,5-diphenyltetrazolium bromide (MTT) assay. (**A**) Nitrite production, (**B**) cell viability, and (**C**) IC_50_ value of fractions to inhibit NO production. (**D**) Western blot analysis of BV2 cells treated with each fraction and activated with LPS. Tubulin was used as a loading control. (**E**) Reactive oxygen species (ROS) production was measured by DCFDA (2’,7’-dichlorodihydrofluorescein diacetate) treatment after 2 h of LPS activation and sample treatment. Scale bars = 50 μm. * *p* < 0.05, ** *p* < 0.01 and *** *p* < 0.001 indicate significant differences compared with treatment of LPS alone, while ## *p* < 0.01 and ### *p* < 0.001 indicate significant differences compared with the untreated control group. Ctl indicates untreated control cells, LPS indicates LPS-treated cells.

NO is a key biomarker of neuroinflammation, wherein its production is used for screening anti-inflammatory potency of samples. In this study, we treated BV2 cells with different fractions of *A. holophylla*, namely, butanol (JGB), chloroform (JGC), ethyl acetate (JGE), and hexane (JGH) fractions. Among these, JGC, JGE, and JGH showed a sharp decline in the NO production in a concentration-dependent manner, while JGB showed moderate inhibition that was not significant (Figure 1A). Higher concentrations (50 µg/mL) of JGC and JGH showed toxicity when treated for 24 h; however, no cytotoxicity was observed up to 6 h of treatment. Hence, we treated BV2 cells for 6 h with 50 µg/mL of the fractions to check the protein expression of iNOS and COX-2. Similar to its effect on NO production, JGB did not significantly inhibit iNOS expression; however, JGC, JGE, and JGH significantly inhibited iNOS expression. Interestingly, all the fractions showed high potency to lower COX-2 expression, of which JGC and JCH showed higher inhibition (Figure 1D). In addition, these fractions inhibited ROS production, wherein JGC and JGH showed higher potency than JGB and JGE (Figure 1E).

### 2.1. A. holophylla Subfractions Inhibited MAPK Signaling and Production of Pro-Inflammatory Cytokines in LPS-Activated Microglia

**Figure 2:**
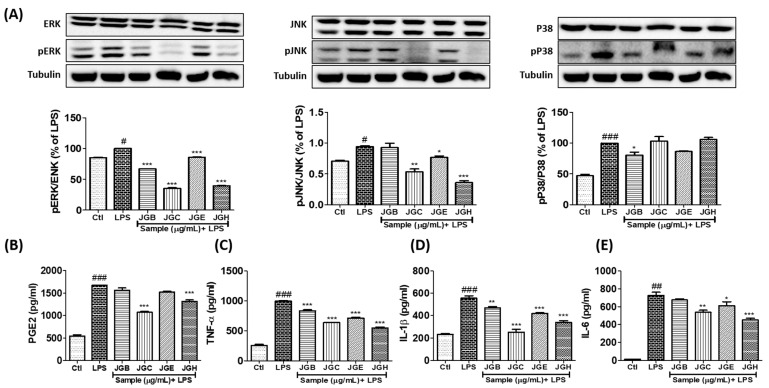
*A. holophylla* subfractions controlled mitogen-activated protein kinase (MAPK) protein expression and secretion of pro-inflammatory cytokines and mediators. Western blot analysis of BV2 cells pre-treated with the JGB, JGC, JGE, and JGH fractions and activated with LPS. (**A**) Expression of MAPKs and their quantification using ELISA. Tubulin was used as a loading control. BV2 cells were treated with 10 μg/mL JGB, JGC, JGE, and JGH and incubated with 100 ng/mL LPS for 24 h; secreted inflammatory mediators were measured using ELISA. (**B**–**E**) Amount of secreted prostaglandin E2 (PGE2), tumor necrosis factor alpha (TNF-α), interleukin (IL)-1β, and IL-6. * *p* < 0.05, ** *p* < 0.01, and *** *p* < 0.001 indicate significant differences compared with treatment of LPS alone, while # *p* < 0.05, ## *p* < 0.01 and ### *p* < 0.001 indicate significant differences compared with the untreated control group. Ctl indicates untreated control cells, LPS indicates LPS-treated cells.

Inhibition of pro-inflammatory mediators induced by *A. holophylla* subfractions was mediated by regulating MAPK signaling. JGC and JGH dramatically lowered phosphorylation of JNK and ERK. JGB and JGE significantly inhibited ERK phosphorylation; however, only JGE inhibited JNK phosphorylation. Among all the fractions, only JGB could downregulate p38 phosphorylation. Moreover, JGC and JGH inhibited the production of PGE2, TNF-α, IL-1β, and IL-6. Although JGB and JGE did not show significant effect on inhibition of PGE2 production, they significantly inhibited TNF-α and IL-1β production. JGE but not JGB inhibited IL-6 production. These results suggest that the JGC and JGH fractions of *A. holophylla* mediated inhibition of MAPK by inhibiting production of the inflammatory mediator (PGE2) and pro-inflammatory cytokines (TNF-α, IL-1β, IL-6). (Figure 2).

### 2.2. Active Components from A. holophylla Subfractions Inhibited Inflammatory NO Production in LPS-Activated Microglia

**Figure 3:**
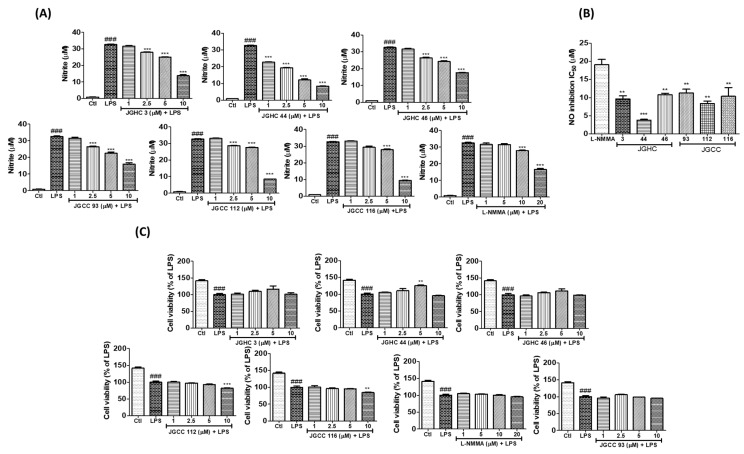
Active components from *A. holophylla* subfractions inhibited nitrite production without exhibiting cytotoxicity. BV2 cells were pre-treated with indicated concentrations of *A. holophylla* phytochemicals JHC 3, JGHCC 44, JGHC 46, JGCC 93, JGCC 112, and JGCC 116 followed by 100 ng/mL LPS activation for 24 h and nitrite production; cell viability was measured. (**A**) Phytochemicals inhibited nitrite production. (**B**) IC_50_ values of phytochemicals that inhibited NO production. (**C**) Cell viability after phytochemical treatment in LPS-activated microglia. ** *p* < 0.01, and *** *p* < 0.001 indicate significant differences compared with treatment of LPS alone, while ### *p* < 0.001 indicates significant differences compared with the untreated control group. Ctl indicates untreated control cells, LPS indicates LPS-treated cells.

On observing that the fractions of *A. holophylla*, especially JGC and JGH, significantly inhibited NO production, we evaluated whether the phytochemicals isolated from JGC and JGH would exhibit similar inhibitory effects. We have previously reported that firmanoic acid (JGHC 3), 23-oxomariesic acid A (JGHC 46), and 7,14,24-mariesatrien-26,23-olide-3*α*,23-diol (JGHC 44) are the main compounds isolated from the hexane fraction, and 23-hydroxy-3-oxolanosta-8,24-dien-26,23-olide (JGCC 93), mangiferolic acid (JGCC 112), and mangiferonic acid (JGCC 116) are the main compounds isolated from the chloroform fraction [12]. All the isolated phytochemicals significantly inhibited NO production in a dose-dependent manner. Among them, JGHC 44 and JGCC 112 showed the highest potency. IC_50_ value of all the phytochemicals was significantly lower than that of the positive control NG-mono-methyl-l-arginine (l-NMMA). However, 10 µM of JGCC 112 and JGCC 116 showed significant toxicity, whereas other phytochemicals did not show significant toxicity up to a concentration of 10 µM (Figure 3).

### 2.3. Active Components from A. holophylla Subfractions Inhibited Inflammatory Protein Expression in LPS-Activated Microglia

**Figure 4:**
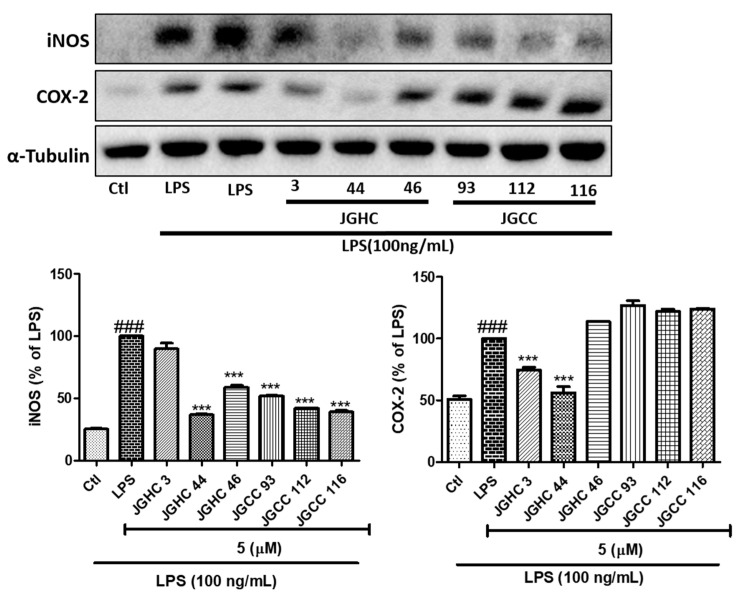
Active components from *A. holophylla* subfractions inhibited expression of inducible nitric oxide synthase (iNOS) and cyclooxygenase (COX-2) in LPS-activated microglia. Western blot analysis of BV2 cells pre-treated with the JHC 3, JGHCC 44, JGHC 46, JGCC 93, JGCC 112, and JGCC 116 subfractions and activated with LPS. Expression and quantification of iNOS and COX-2 against LPS-activated microglia. *** *p* < 0.001 indicates significant differences compared with treatment of LPS alone, while ### *p* < 0.001 indicates significant differences compared with the untreated control group. Ctl indicates untreated control cells, LPS indicates LPS-treated cells.

Phytochemical-mediated inhibition of NO production was confirmed by the reduced protein expression of iNOS by all subfractions. However, only JGHC 3 and JGHC 44 inhibited the expression of COX-2 (Figure 4). As NO, iNOS, and COX-2 are inflammatory biomarkers of M1 microglia, inhibition of these biomarkers suggests the role of isolated phytochemicals in lowering activated microglia-mediated neuroinflammation.

### 2.4. Active Components from A. holophylla Subfraction Inhibited ROS Production and MAPKs in LPS-Activated Microglia

**Figure 5:**
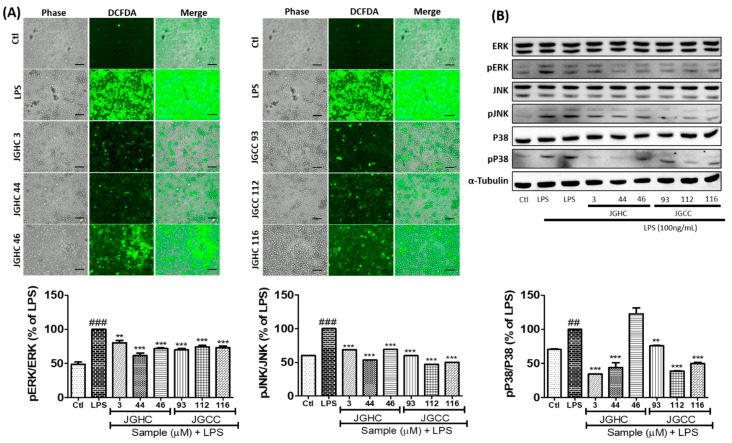
Active components from *A. holophylla* subfractions inhibited ROS production and MAPK expression. BV2 cells were pre-treated with 5 μM of JHC 3, JGHCC 44, JGHC 46, JGCC 93, JGCC 112, and JGCC 116, and activated with LPS (100 ng/mL) for 30 min; cells were incubated with DCFDA for measuring ROS production. Treated cells were harvested and protein expression was observed using Western blot analysis. (**A**) ROS production. Scale bars = 50 μm. (**B**) Western blot analysis and quantification of MAPK signaling proteins. Tubulin was used as a loading control. ** *p* < 0.01 and *** *p* < 0.001 indicate significant differences compared with treatment of LPS alone, while ## *p* < 0.01 and ### *p* < 0.001 indicate significant differences compared with the untreated control group. Ctl indicates untreated control cells, LPS indicates LPS-treated cells.

Phytochemicals from *A. holophylla* inhibited ROS production in LPS-activated microglia, suggesting their possible antioxidant properties that can play a pivotal role in downregulating inflammatory cascades (Figure 5A). Additionally, the isolated compounds showed great potency to downregulate LPS-activated MAPK signaling. Phosphorylation of ERK and JNK was significantly inhibited by all phytochemicals. Except JGHC 46, all phytochemicals significantly inhibited phosphorylation of p38 (Figure 5B). JGHC 44 showed the highest potency to inhibit phosphorylation of ERK, JNK, and p38 in activated microglia, suggesting that JGHC 44 could be the lead compound in *A. holophylla* possessing an anti-neuroinflammatory property.

### 2.5. Active Components from A. holophylla Fractions Inhibited PGE2 and TNF-α Production in LPS-Activated Microglia

**Figure 6:**
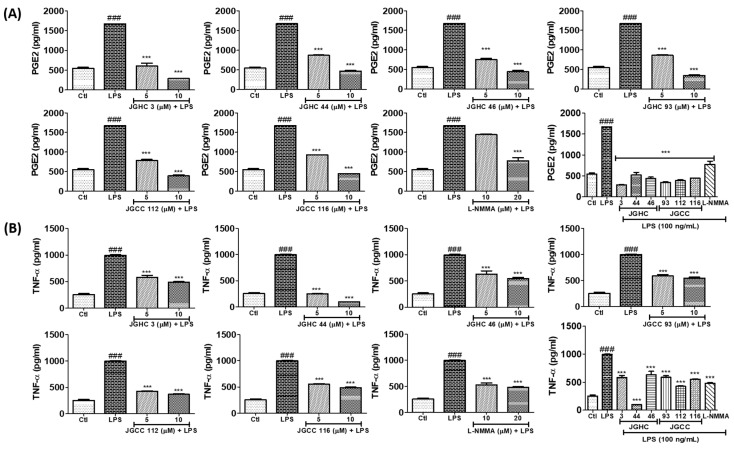
Active components from *A. holophylla* subfraction inhibited PGE2 and TNF-α-production against LPS-activated microglial cells. Measurement of pro-inflammatory mediators in BV2 cells pre-treated with the JHC 3, JGHCC 44, JGHC 46, JGCC 93, JGCC 112, and JGCC 116 subfractions and activated with LPS. In addition, the conditioned medium was used for this analysis. NG-mono-methyl-l-arginine (l-NMMA) was the positive control. (**A**) Effect on PGE2 production and (**B**) TNF-α production. *** *p* < 0.001 indicates significant differences compared with treatment of LPS alone, while ### *p* < 0.001 indicates significant differences compared with the untreated control group. Ctl indicates untreated control cells, LPS indicates LPS-treated cells.

**Figure 7:**
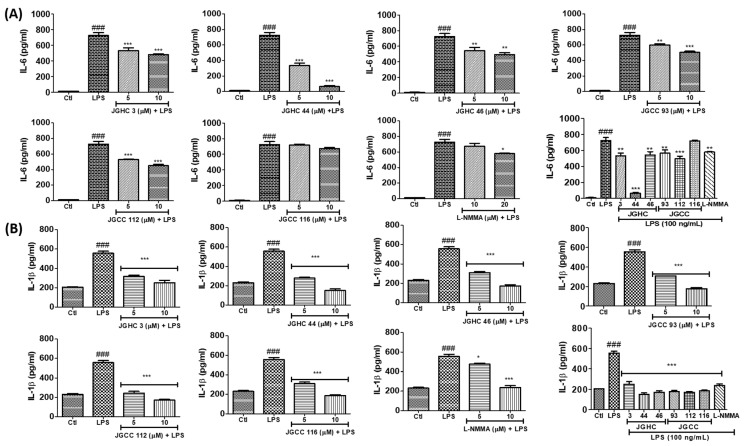
Active components from *A. holophylla* subfraction inhibited the production of IL-6 and IL-1β against LPS-activated microglial cells. BV2 cells were pre-treated with 5 μM of JHC 3, JGHCC 44, JGHC 46, JGCC 93, JGCC 112, and JGCC 116, and 20 μM l-NMMA (positive control) and activated with LPS (100 ng/mL) for 24 h; cells and conditioned medium were used for the measurement of pro-inflammatory cytokines. (**A**) Effect on IL-6 production and (**B**) IL-1β production. * *p* < 0.05, ** *p* < 0.01, and *** *p* < 0.001 indicate significant differences compared with treatment of LPS alone, while ### *p* < 0.001 indicates significant differences compared with the untreated control group. Ctl indicates untreated control cells, LPS indicates LPS-treated cells.

All the phytochemicals markedly inhibited PGE2 production, whereby this potency was much higher than that of the positive control L-NMMA (Figure 6A). Additionally, all the isolated phytochemicals inhibited production of the pro-inflammatory cytokines TNF-α, IL-6, and IL-1β to varying extents (Figure 7A,B). JGHC 44 (from the hexane fraction) and JGCC 112 (from the chloroform fraction) almost normalized the production of TNFα against LPS-activated cells to a level similar to that of the untreated control group. Similarly, all except JGHC 116 significantly inhibited IL-6 production. Interestingly, all the phytochemicals significantly inhibited IL-1β in LPS-activated microglia. However, JGHC 44 showed the highest potency in inhibiting pro-inflammatory cytokines in LPS-activated microglia, and its inhibitory effect was greater than that of l-NMMA. These results suggest that the phytochemicals, especially JGHC 44 from *A. holophylla*, have strong anti-inflammatory potency.

## 3. Materials and Methods

### 3.1. Reagents

LPS, MTT (3-(4,5-dimethylthiazol-2-yl)-2,5-diphenyltetrazolium bromide), and dichlorofluorescein diacetate (DCFH-DA) were purchased from Sigma-Aldrich (St. Louis, MO, USA). Enzyme-linked immunosorbent assay (ELISA) kits for TNF-α, IL-6, IL-1β, and PGE2 were obtained from R&D Systems (Minneapolis, MN, USA). Dulbecco’s modified Eagle’s medium (DMEM), penicillin–streptomycin, and fetal bovine serum (FBS) were purchased from Invitrogen (Carlsbad, CA, USA). Primary antibodies for iNOS, COX-2, β-actin, JNK, pJNK, p38, pp38, ERK, pERK, and α-tubulin were purchased from Cell Signaling Technology (Danvers, MA, USA). Pro-prep solution was obtained from iNtRON Biotechnology (Seoul, South Korea). ECL Western Blotting Detection Reagents were purchased from Amersham Pharmacia Biotech (Buckinghamshire, UK). Purified LPS from *Esherichia coli* was purchased from Sigma (St. Louis, MO, USA).

### 3.2. Extraction and Isolation of A. hollophylla

**Figure 8:**
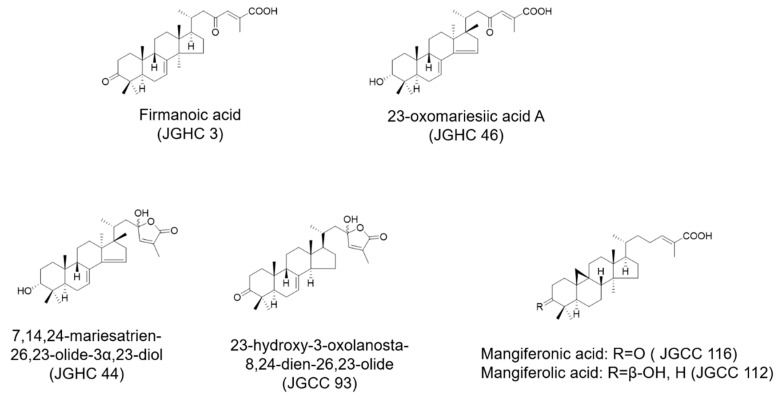
Structures of terpenoids isolated from chloroform- and hexane-soluble fractions of *A. hollophylla*.

*A. hollophylla* were extracted and isolated as described in our previous study [11]. Briefly, the *A. hollophylla* trunk was extracted using 80% MeOH (methanol). The MeOH extract was further partitioned with n-hexane, chloroform, ethyl acetate, and n-butanol. The n-hexane soluble fraction was separated using a silica gel column (CHCl_3_-MeOH, 70:1) while the chloroform soluble fraction was separated using a silica gel column (CHCl_3_-MeOH, 50:1) to yield their respective fractions. The fractions were further purified using semipreparative HPLC. The purified n-hexane and chloroform fractions are shown in Figure 8.

Murine microglial cells (BV2) used in the present study were received from Korea University (Seoul, Korea). The cells were maintained in DMEM with high glucose supplemented with 10% heat-inactivated FBS and 1% penicillin (1 × 10^5^ U/L)-streptomycin (100 mg/L) in a humidified incubator with 5% CO_2_ at 37 °C.

### 3.3. Cell Treatment, Nitric Oxide Production, and Cell Viability Assay

Cell viability was measured using the MTT colorimetric assay. BV2 cells were seeded in a 96-well plate at a density of 4 × 10^4^ cells per well and incubated for 24 h. Pre-treatment of extracts, compounds, and inhibitors was performed 30 min before activating the cells with LPS (100 ng/mL). After 24 h of treatment, 50 μL of conditioned medium was transferred to a new plate and mixed with an equal volume of Griess reagent (1% sulfanilamide in 5% phosphoric acid and 0.1% *N*-(1-naphthylethylenediamine dihydrochloride)). The presence of NO resulted in the development of pink color, and the change in color was quantified by measuring the absorbance at 540 nm in a microplate reader. Sodium nitrite (NaNO_2_) was used as the standard. NG-mono-methyl-l-arginine (l-NMMA), an NOS (nitric oxide synthase) inhibitor, was used as positive control (20 μM). In the original plate, the remaining conditioned medium was removed, and MTT solution (0.5 μg/mL) was added. The plate was incubated under dark conditions for approximately 1 h in a humidified incubator with 5% CO_2_ at 37 °C. Consequently, viable cells converted yellow MTT to violet-blue formazan. To solubilize the resulting water-insoluble formazan, we removed the MTT solution and added approximately twice the volume of dimethyl sulfoxide (DMSO) to the wells. Absorbance was measured at 570 nm using a microplate reader (VersaMax, USA). Cell viability was calculated considering the LPS-treated group as 100%.

### 3.4. Measurement of PGE2, TNF-α, IL-1β, and IL-6 Production

For measuring pro-inflammatory cytokines, we seeded BV2 cells in a 6-well plate at a density of 1.5 × 10^6^ cells per well and incubated for 24 h. Cells were pre-treated with *A. holophylla* soluble fractions and its subfraction and exposed with or without LPS and incubated for the next 24 h. Conditioned medium from the treated cells was collected and stored at −80 °C until further use (this medium can be stored for up to a few months for ELISA). ELISA for TNF-α, IL-1β, and IL-6 was performed using ELISA development kits of the particular cytokine, while PGE2 was measured using a competitive enzyme immunoassay kit (Cayman Chemical, Ann Arbor, MI, USA). All experiments were performed as per the protocol provided with the respective ELISA kit.

### 3.5. Western Blot Analysis

For evaluating protein expression, we performed Western blot analysis of the cell lysate. Cells were seeded and treated with different required time according to the location and time of activation. Next, the cells were harvested and lysed with Pro-prep (iNtRON Biotechnology, Seoul, South Korea) solution supplemented with proteinase and phosphatase inhibitors according to the requirement. After total protein extraction from the cell lysate, we performed protein estimation using Bradford assay with bovine serum albumin (BSA) as the standard. Absorbance was measured at 595 nm using a microplate reader. A total of 30 μg of protein was used to separate the desired protein using SDS-PAGE, following which the gel was transferred to nitrocellulose membrane. Next, the membranes were incubated with primary antibodies against tubulin, iNOS, COX-2, ERK, pERK, JNK, pJNK, p38, pp38, and α-tubulin, and incubated overnight at 4 °C. After overnight incubation with the primary antibody, membranes were washed and incubated with horseradish peroxidase-conjugated secondary antibodies, and protein bands were visualized using ECL Western Blotting Detection Reagents. Densitometry analysis of the bands was performed using Image Master 2D Elite software (v. 3.1, Amersham Pharmacia Biotech).

### 3.6. Measurement of ROS Production

BV2 cells were seeded in a 6-well plate and treated with DCFH-DA for 30 min prior to LPS activation. After activation, the cells were incubated for 2 h. Following incubation, 20 µM DCFH-DA dye was added to each well and the plate was incubated for 30 min. Next, the cells were washed with 1X PBS (phosphate buffer saline), and 1 mL 1X PBS was added to each well. Stained cells were analyzed for green florescence and photographed under a phase contrast microscope (JuLI live-cell imaging system, NanoEnTek, Seoul, Korea). Images were taken in several fields randomly.

### 3.7. Statistical Analysis

Experimental results are presented as mean ± standard error of the mean (SEM). Statistical analysis between the test groups was performed by one-way analysis of variance (ANOVA) followed by the Newman–Keuls post hoc test using GraphPad Prism 5 (GraphPad Software Inc., La Jolla, CA, USA). Statistical significance was set at *p* < 0.05.

## 4. Discussion

Neuroinflammation is associated with the pathology of numerous neurological complications, including AD, PD, MS (multiple sclerosis), and ischemia [1,13,14]. Neuroinflammation-mediated neurodegeneration is the key molecular event responsible for the initiation and severity of such diseased conditions [3]. Effective treatment strategies include lowering neuroinflammation or preventing neurodegeneration. Microglial activation to inflammatory phenotype, which results in overproduction of inflammatory mediators, is the key player in pathogenesis of neurodegeneration and neurotoxicity [15,16]. A number of phytochemicals have been reported to exhibit strong potency to lower microglial activation as well as production of inflammatory mediators [17]. These phytochemicals are being considered as possible candidates for the maintenance of normal CNS homeostasis by counteracting neuroinflammation and neurodegeneration. Microglia are responsible for host defense against pathogens. Activated microglia release various pro-inflammatory cytokines and ROS, often causing neuronal cell degeneration [18]. Previously, we reported that diterpenes isolated from *A. holophylla* have anti-inflammatory effects in LPS-activated murine microglial cells (via reduced NO production) and neuroprotective properties in C6 glioma cells (via increased nerve growth factor secretion) [11,12]. In the present study, we explored the anti-neuroinflammatory properties of *A. holophylla* extracts and its active phytochemicals on neuroinflammatory events induced by LPS-activated microglia in the murine microglial cells, BV2. COX-2 is a biomarker of inflammatory M1 microglial phenotype, whereby its overexpression is associated with acute inflammation [19]. LPS, proinflammatory cytokines, and growth factors induce COX-2 expression; this activation of COX-2 controls the synthesis of PGE2 [20]. Previous studies have indicated that NO and iNOS overproduction increases oxidative stress and is related to various neurodegenerative diseases [6,21,22]. Additionally, the release of proinflammatory cytokines (TNF-α, IL-1β, and IL-6) by microglia is increased during neuroinflammation [23,24]. In our study, chloroform- and hexane-soluble fractions of *A. holophylla* and their active compounds significantly inhibited the LPS-induced ROS and NO production with increased COX-2 and PGE2 expression.

Many studies have reported that MAPK is the key signaling mechanism responsible for controlling inflammatory pathways in LPS-activated microglia [25,26,27]. Activation of the MAPK signaling pathway induces the release of pro-inflammatory cytokines, including IL6, IL-1β, and TNF-α, as a secondary response [25]. JNK is a stress-induced kinase that is activated during inflammation. Many studies have indicated the activation of the JNK pathway that induces the release of cytokines in LPS-activated microglial cells [28]. Inhibition of JNK signaling suppresses the secretion of IL6, IL-1β, and TNF-α that results in the impediment of inflammation [29,30]. In agreement with the previous results, chloroform- and hexane-soluble fractions of *A. holophylla* and their active compounds significantly inhibited the LPS-induced activation JNK, thereby suppressing the secretion of IL6, IL-1β, and TNF-α. In addition to JNK, p38 plays a central role in inflammation. The p38 pathway is responsible for regulating pro-inflammatory cytokines [31]. Both in vivo and in vitro studies have indicated that inhibition of the p38 and JNK pathways plays a significant role in suppressing inflammation [6,22,26,31,32,33]. The anti-neuroinflammatory effects of all the active components from *A. holophylla* were higher than those of L-NMMA, an iNOS inhibitor. Among the phytochemicals, JGHC 44 showed the highest potency of inhibiting phosphorylation of JNK and p38, with further inhibition on the release of pro-inflammatory cytokines (IL6, IL-1β, TNF-α) and mediators COX-2 and PGE2. These results indicate that the hexane-soluble fraction of *A. holophylla* and its active compound 7,14,24-mariesatrien-26,23-olide-3*α*,23-diol (JGHC 44) were mainly responsible for the anti-neuroinflammatory effect of *A. holophylla* on LPS-activated murine microglial cells. Our study demonstrates the anti-neuroinflammatory mechanism of *A. holophylla* and its active components for the first time.

## 5. Conclusions

In conclusion, chloroform- and hexane-soluble fractions of *A. holophylla* significantly inhibited the inflammatory mediators in LPS-activated murine microglia, suggesting that this medicinal plant is a potential candidate for drug discovery and futuristic treatment of neuroinflammation. Triterpenoids from *A. holophylla* subfraction, 7,14,24-mariesatrien-26,23-olide-3*α*,23-diol (JGHC 44), have excellent potency to regulate MAPK signaling and inflammatory cytokines in BV2 cells. Our study provides a great insight for the development of appropriate therapeutics against neurological complications, especially where cytotoxicity and pathogenesis are mediated by activated microglia. Further research on *A. holophylla* and its active components at the cellular, molecular, and in vivo levels can reveal its potential therapeutic application in inflammatory conditions in general (that is, not limited to neuroinflammation). We consider that among the screened phytochemicals, further attention to JGHC 44 can unravel its potency against neuroinflammation, as this compound showed evident inhibitory effects even at lower concentrations. Either the compound as a whole or its structurally modified derivatives could be better candidates for the treatment of neurological complications in future.

## Data Availability

All data generated or analyzed during this study are included in this published article.
